# Multidisciplinary Treatment Strategies for Wilms Tumor: Recent Advances, Technical Innovations and Future Directions

**DOI:** 10.3389/fped.2022.852185

**Published:** 2022-07-14

**Authors:** Till-Martin Theilen, Yannick Braun, Konrad Bochennek, Udo Rolle, Henning C. Fiegel, Florian Friedmacher

**Affiliations:** ^1^Department of Pediatric Surgery and Pediatric Urology, University Hospital Frankfurt, Goethe University Frankfurt, Frankfurt, Germany; ^2^Division of Pediatric Hematology and Pediatric Oncology, Hospital for Children and Adolescents, University Hospital Frankfurt, Goethe University Frankfurt, Frankfurt, Germany

**Keywords:** nephroblastoma, kidney neoplasm, surgical oncology, nephron sparing surgery, minimal invasive surgery, nephrectomy, therapy, biomarkers

## Abstract

Significant progress has been made in the management of Wilms tumor (WT) in recent years, mostly as a result of collaborative efforts and the implementation of protocol-driven, multimodal therapy. This article offers a comprehensive overview of current multidisciplinary treatment strategies for WT, whilst also addressing recent technical innovations including nephron-sparing surgery (NSS) and minimally invasive approaches. In addition, surgical concepts for the treatment of metastatic disease, advances in tumor imaging technology and potentially prognostic biomarkers will be discussed. Current evidence suggests that, in experienced hands and selected cases, laparoscopic radical nephrectomy and laparoscopic-assisted partial nephrectomy for WT may offer the same outcome as the traditional open approach. While NSS is the standard procedure for bilateral WT, NSS has evolved as an alternative technique in patients with smaller unilateral WT and in cases with imminent renal failure. Metastatic disease of the lung or liver that is associated with WT is preferably treated with a three-drug chemotherapy and local radiation therapy. However, surgical sampling of lung nodules may be advisable in persistent nodules before whole lung irradiation is commenced. Several tumor markers such as loss of heterozygosity of chromosomes 1p/16q, 11p15 and gain of function at 1q are associated with an increased risk of recurrence or a decreased risk of overall survival in patients with WT. In summary, complete resection with tumor-free margins remains the primary surgical aim in WT, while NSS and minimally invasive approaches are only suitable in a subset of patients with smaller WT and low-risk disease. In the future, advances in tumor imaging technology may assist the surgeon in defining surgical resection margins and additional biomarkers may emerge as targets for development of new diagnostic tests and potential therapies.

## Introduction

Renal solid tumors account for ~5% of all childhood tumors with 8.3–15.1 cases per one million person-years worldwide (1–3). Over 90% of these renal malignancies are Wilms tumors (WT; syn. nephroblastoma) presenting at a peak incidence of 2–3 years of age (4). The majority of cases are sporadic, whereas 10–15% present in relation to genetic malformation syndromes predisposing to tumor development such as Beckwith-Wiedemann, Denys-Drash or WAGR (i.e., WT, aniridia, genitourinary anomalies and range of intellectual disabilities) (5).

The first successful nephrectomy for a WT in a 2-year-old boy by Thomas Richard Jessop in 1877 (Leeds, England), the first histopathological description of WTs by “Max” Wilms in 1899 (Leipzig, Germany) as well as the introduction of radiation therapy and cytotoxic chemotherapy in the 1950s and 1960s are the foremost origins of what has led to today's treatment standards for WT patients (6–8). Collaborative studies by the National Wilms Tumor Study Group (NWTS)/Renal Tumor Committee of the Children's Oncology Group (COG) in North America and by the International Society of Pediatric Oncology (SIOP) in Europe laid the groundwork for protocol-driven treatment plans. Today, a combination therapy of surgical resection, adjuvant and/or neoadjuvant chemotherapy and in some cases irradiation achieves an overall survival (OS) exceeding 90% for localized WT, 75% for metastasized WT and 50% in the case of WT recurrence (9).

Despite the good outcome for the majority of patients with WT, some remain at risk for poor survival or have a treatment related long-term risk of side effects (10). Risk factors for reduced event-free survival (EFS) and OS are: more than two-third blastemal cells within the tumor after induction chemotherapy (SIOP protocol, 8–9% of patients), anaplastic histology (5–10% of patients), loss of heterozygosity (LOH) at chromosomes 1p/16q (12 and 17% of patients, respectively), (non-resolving) metastasis (12–17% of patients), bilateral WT (5–8% of patients) and recurrent WT (20% of patients) (11–19). These patients need intensive chemotherapy and radiation therapy, thus putting them at risk for treatment related side effects. Overall, 25% of WT patients suffer from subsequent side effects such as renal failure, cardiac toxicity, pulmonary restrictive disease, infertility and secondary malignancies (20, 21).

Recent and ongoing clinical trials drive treatment strategies toward a more and more risk-based and individualized therapy approach. The identification of personal risk factors will eventually confine intensive therapy to smaller subgroups of patients, thus reducing chemotherapy-related and radiation-related side effects for others without reducing survival.

This article provides a comprehensive overview of current multidisciplinary treatment strategies for WT, whilst also addressing recent technical innovations including nephron-sparing surgery (NSS) and minimally invasive approaches. In addition, surgical concepts for the treatment of metastatic disease, advances in tumor imaging technology and potentially prognostic biomarkers will be discussed.

## Current Diagnostic Work-Up and Staging

The incidental palpation of an asymptomatic abdominal mass is the most common presentation of a child with WT. In only 20% of cases, the presentation consists of malaise, pain, fever, gross hematuria or renal hypertension (22, 23). Rarely, an acute abdomen due to tumor rupture is the first presentation of a WT. In other cases, WT is diagnosed by routine ultrasonography in patients with conditions predisposing for WT such as Beckwith-Wiedemann syndrome or in patients in whom metachronous metastasis is suspected.

Abdominal ultrasonography is the first diagnostic choice to confirm a renal mass. To differentiate a WT from other renal masses (e.g., kidney malformations) or masses in close proximity to the kidney (e.g., neuroblastoma), abdominal MRI- and CT-scans are the current standards of imaging. Urine analysis for catecholamines and metaiodobenzylguanidine scintigraphy can also help to discriminate neuroblastoma from WT. Further work-up includes laboratory tests screening for tumor-associated anemia and thrombocytopenia, kidney malfunction, altered liver enzymes in case of liver metastasis and disrupted coagulation such as WT-acquired von Willebrand disease.

In the NWTS-4, patients with a diagnostic biopsy had a higher risk for local recurrence leading to an upgrading of a stage I and II tumor to a stage III tumor in later protocols (24). An upstaging is not recommended in the SIOP protocol, as studies have not confirmed local recurrence (except in case of open tumor biopsy) (25). In general, biopsies should only be considered when a tumor different to WT is suspected. This is usually the case when renal tumors present at an age older than 6 years, the mass is completely extra renal or imaging shows distinct calcifications suggesting a histology other than WT (26).

MRI, CT and ultrasound scans contribute to pretreatment staging. The contralateral kidney should be screened for synchronous bilateral disease. Aortocaval and hilar lymph nodes, lung and liver need to be screened for metastasis. CT scans (with or without contrast) have replaced plain X-ray images of the chest to detect lung metastasis (27–30). Contrast MRI (or CT) and ultrasound examinations will need to assess extension of the tumor into the renal vein and vena cava. Echocardiography is warranted to assess heart function and the venous extension of a tumor thrombus into the atrium (31–33).

Current staging systems of WT are based on imaging and surgical findings such as local tumor stage (e.g., complete vs. incomplete resection, perioperative tumor rupture or lymph node metastasis), bilateral disease and hematogenous metastasis to the lung and liver (rarely bone, brain or other sites) (Table 1). The histopathology of the resected tumor defines the prognostic risk group. The NWTS/COG classifies tumors in favorable and unfavorable, whereas SIOP differentiates low-, intermediate- and high-risk tumor histopathology according to the predominant cell components within the tumor (30, 34, 35) (Table 2).

**Table 1:** Current staging systems of WT according to NWTS/COG and SIOP (post-surgery).

**Stage**		**NWTS/COG staging**	**SIOP staging**
I	Complete resection with negative margins	Primary tumor within renal capsule, no capsule involvement No extension to renal sinus	Tumor is confined to the kidney, no penetration of the renal capsule No tumor infiltration of the vessels at the renal sinus, the renal pelvis and the ureter
II	Complete resection with negative margins	Primary tumor penetrating renal capsule but not Gerota's fascia Lymphatic and venous involvement at renal sinus	Viable tumor is present within – soft tissue of renal sinus – the wall of renal pelvis or ureter
		Tumor extension into renal vein/vena cava	Viable tumor is present but completely resected within – perirenal fat – blood vessels/lymphatic vessels at the renal sinus – adjacent organs (except adrenal gland) – vena cava
III	Incomplete resection, residual tumor	Macroscopic or microscopic residual disease – Preoperative biopsy of the tumor (including open and percutaneous biopsy) – Tumor rupture pre- or intraoperatively, removal of tumor tissue in fragments – Positive retroperitoneal/abdominal lymph node(s) – Peritoneal involvement – Positive resection margins at ureter, renal vein, main tumor	Viable tumor – at the resection margin – at the site of tumor rupture Viable or non-viable tumor – in abdominal lymph nodes – thrombus at the resection margin of ureter, renal vein, vena cava – implants within the abdominal cavity – penetrating through the peritoneal surface
			Venous tumor thrombus resected piecemeal Open tumor biopsy prior to chemotherapy
IV	Metastatic disease	Hematogenous metastasis (e.g., lung, liver, bone or brain)	Hematogenous metastasis (e.g., lung, liver, brain or bone) Lymphatic metastasis beyond abdominal and retroperitoneal lymph nodes
V	Bilateral disease	Bilateral synchronous disease (+ stage should be evaluated for each side separately)	Bilateral synchronous disease (+ stage should be evaluated for each side separately)

**Table 2:** Prognostic risk groups for WT according NWTS/COG and SIOP relating to the histopathology of embryonal renal tumors in childhood.

**NWTS/COG**		**SIOP**	
**Prognostic risk group**	**Histology (pre-chemotherapy)**	**Prognostic risk group**	**Histology (post-chemotherapy)**
Mesoblastic histology	Mesoblastic nephroma^*^	Low-risk	Mesoblastic nephroma^*^ Cystic partially differentiated nephroblastoma^*^ Completely necrotic WT after neoadjuvant chemotherapy
Favorable histology	WT with – epithelial – stromal – blastemal	Intermediate- risk	WT with – epithelial – stromal – or mixed cell components
	– or mixed (triphasic) cell components		Regressive histology features Focal anaplasia
Unfavorable histology	WT with focal and diffuse anaplasia	High-risk	WT with – blastemal cells – diffuse anaplasia
			Renal rhabdoid tumor^*^ Renal clear cell sarcoma^*^ Renal cell carcinoma^*^

* *Non-Wilms tumors*.

In recent years, central review panels assist in staging, radiology interpretation, surgical decision-making and histology examinations of patients with WT [established by the NWTS/COG (i.e., AREN03B2 umbrella study) as well as by the SIOP-Renal Tumor Study Group (RTSG) (i.e., UMBRELLA SIOP-RTSG 2016; https://fnkc.ru/docs/SIOP-RTSG2016.pdf)] (30, 34, 36). It has been shown that the COG central review adjusted the initial risk stratification in ~20% of cases (23). Centralized review and assistance by WT study groups may eventually lead to more concise outcome data. To integrate and combine the complex sets of medical data, e-health tools have been developed such as “p-medicine” utilized by the UMBRELLA SIOP-RTSG protocol.

## Overview of Current Multidisciplinary Treatment Strategies

The two most commonly applied treatment regimens for WT derive from the NWTS/COG and SIOP. Current treatment protocols of both groups are based on the concept of risk-adapted therapy including surgery, chemotherapy and irradiation. Throughout the multiple WT studies, patients have been identified as requiring either intensified or reduced therapy according to their individual risk factors. The NWTS/COG-based protocols have followed the concept of upfront tumor resection to plan therapy according to biological information and local stage of the tumor. In contrast, the SIOP protocol includes upfront chemotherapy before surgical resection to shrink and downstage the tumor, thus increasing the chance for complete resection and minimizing the operative risk of a tumor rupture. However, in case of a kidney tumor in a child under 6 months of age, the SIOP protocol recommends upfront tumor resection, as most kidney tumors in this age group either need no further therapy (e.g., congenital mesoblastic nephroma) or need intensified chemotherapy at the outset (e.g., malignant rhabdoid tumors) (30). Local stage and tumor biology findings are used for a risk-modified treatment. Despite these strategic treatment differences between the NWTS/COG and SIOP approach, patient outcome is nearly identical.

### Metastatic Disease

About 10–12% of patients with WT present with metastasis. Lymph node, lung and liver are the most prominent metastatic sites. In recent studies, particular attention has been given to the treatment of lung metastasis in WT. Standard treatment for lung metastasis has consisted of escalated systemic therapy and whole lung radiation therapy (WLRT) regardless of lung nodule response. In the NWTS/COG AREN0533 study, patients with complete response in chest CT scans after 6 weeks of DD-4A did not receive further WLRT. However, patients with incomplete response and those with LOH at chromosomes 1p/16q received additional intensified therapy with four cycles of systemic cyclophosphamide and etoposide and WLRT according to the NWTS/COG protocol (Box 1). Both groups, complete and incomplete responders, had significantly improved 4-year EFS and OS estimates (85.4 and 95.6%, respectively) compared to the previous NWTS-5 (72.5 and 84.0%, respectively) (27). Similar results have been stated by SIOP (17) (Box 2). In uncertain lung lesions on chest CT or in so-called “slow incomplete responders” assessed by chest CT imaging, some studies encourage to biopsy (at least two) such lesions to certify metastasis before WLRT is applied (37).

Box 1 **Basic treatment principles of WT as per NWTS/COG (covering most clinical situations).**

**Stage I (patients** **<2 years of age):** Tumors with favorable histology and tumor kidney weight <550 g: RN without further treatment

**Stage I and II:** Tumors with favorable histology: RN + adjuvant chemotherapy with regimen EE-4A for 18 weeks; tumors with favorable histology and LOH 1p/16q: RN + adjuvant chemotherapy with regimen DD-4A for 24 weeks

**Stage III:** Tumors with favorable histology: RN + adjuvant chemotherapy with regimen DD-4A for 24 weeks + flank RT; tumors with favorable histology and LOH 1p/16q: RN + adjuvant chemotherapy with regimen M for 31 weeks + pulmonary RT

**Stage IV:** Tumors with favorable histology and isolated lung metastasis: RN + adjuvant chemotherapy with regimen DD-4A for 24 weeks + whole-lung RT; tumors with favorable histology and LOH 1p/16q: RN + adjuvant chemotherapy with regimen M for 31 weeks + pulmonary RT

**Stage V:** Neoadjuvant chemotherapy with DD-4A for 6–12 weeks, partial nephrectomy, adjuvant chemotherapy depends on path

LOH, loss of heterozygosity; RN, radical nephrectomy; RT, radiation therapy; regimen EE-4A (vincristine, dactinomycin for 18 weeks); regimen DD-4A (vincristine, dactinomycin, doxorubicin for 24 weeks), regimen M (vincristine, dactinomycin, doxorubicin, cyclophosphamide, etoposide).

Box 2 **Basic treatment principles of WT as per SIOP (covering most clinical situations).**

**Stage I–III:** Neoadjuvant chemotherapy (except patients <6 months of age: RN) with AV for 4 weeks followed by RN, adjuvant therapy is determined by local stage and histology:

– **Stage I:** No further treatment to patients with RN and low-risk histology, all other patients receive chemotherapy with either AV for 4 weeks (intermediate-risk tumors, TV <500ml) or AVD for 27 weeks (high-risk tumors, TV >500ml)
– **Stage II:** Chemotherapy with AV for 27 weeks (low- and intermediate-risk tumors, TV <500ml) or with HR-1 for 34 weeks (high-risk tumors, TV >500ml) + flank RT for high-risk tumors
– **Stage III:** Chemotherapy with AV for 27 weeks (low- and intermediate-risk tumors, TV <500ml) + flank RT for intermediate-risk tumors or with HR-1 for 34 weeks + flank RT (high-risk tumors, TV >500ml)

**Stage IV:** Neoadjuvant chemotherapy with AVD for 6 weeks, re-imaging of metastatic lesions before RN, continuation adjuvant therapy depending on remission of metastasis:

– Complete remission of metastasis: AVD for 27 weeks
– Incomplete response of metastasis: HR-1 for 34 weeks + RT of metastatic organ, surgical resection of metastatic lesions can be attempted when feasible without risk of organ morbidity
– High-risk histology of the primary tumor: HR-1 for 34 weeks + RT of metastatic organ

**Stage V:** Neoadjuvant chemotherapy with AV for 4–6 weeks (12 weeks maximum) followed by surgery with the aim of nephron-sparing surgery, adjuvant chemotherapy according to the histopathological subtype + abdominal/flank RT in appropriate cases.

AV, (actinomycin D, vincristine); AVD, (actinomycin D, vincristine, doxorubicin); HR-1, (etoposide, carboplatin, cyclophosphamide, doxorubicin); low-risk, (i.e., complete necrosis); intermediate-risk, (i.e., blastemal tumor components); high-risk, (i.e., predominant blastemal, anaplastic components); RN, radical nephrectomy; RT, radiotherapy; TV, tumor volume after neoadjuvant therapy.

### Aspects of Surgical Intervention

Complete resection with tumor-free margins remains the primary surgical aim for cure of WT. Generally, the NWTS/COG protocol schedules surgical resection initially after diagnosis before any further treatment. Neoadjuvant chemotherapy is only given to NWTS/COG protocol-treated patients in cases, in which the tumor has ruptured preoperatively, the tumor is deemed to be irresectable (e.g., large tumors with intraoperative risk for rupture, extensive organ invasion with the risk for organ removal and venous tumor extension beyond the hepatic veins) or the patient is at risk for anesthesia-related complications due to cardiocirculatory compromise by a high tumor burden or extensive venous thrombosis. In contrast, SIOP protocol-treated patients are typically operated after four cycles of neoadjuvant chemotherapy.

#### General Surgical Principles

In unilateral WT, complete tumor nephroureterectomy through a transabdominal approach is the standard of surgical care for NWTS/COG- and SIOP-treated patients. The ureter should be resected along the tumor kidney and divided as close as possible to the bladder as the tumor may extend along the ureter. For local staging, hiliar and (inter-)aortocaval lymph node sampling as well as peritoneal exploration is required. Lymph node sampling should be performed even in cases of negative nodal preoperative imaging. An insufficient nodal dissection may lead to under-staging and under-treatment in cases positive lymph nodes remain undetected (24, 38). The impact of an undefined lymph node status in patients with WT has been reviewed by many studies. For instance, NWTS-4 and NWTS-5 both demonstrated an increased likelihood of finding a positive lymph node when more than seven lymph nodes were sampled (39). OS has also been shown to improve significantly with the number of resected lymph nodes (i.e., 5-year OS of 87% with no lymph nodes sampled to 95% with more than 10 lymph nodes sampled) (40). In the current treatment protocols, dissection of at least seven lymph nodes is therefore recommended (30, 34).

The general nature of WT growth is expansion (i.e., pushing organs away). In rare cases, adherence or invasion to adjacent organs such as liver, diaphragm, adrenal gland or bowel is seen. Table 3 summarizes general surgical principles in (unilateral) WT.

**Table 3:** General surgical principles in unilateral WT to achieve local control.

**Organ/Structure**	**Surgical measure**	**Comment**	**References**
Tumor kidney	Radical resection, avoid tumor spillage	Tumor spillage will lead to stage upgrading and more intensive therapy, tumor rupture is associated with relapse	(24, 41)
Ureter	Division at the most distal level (closest to the bladder)	To achieve negative margins in case of tumor involvement of the ureter	(42)
Renal vein, inferior vena cava	Palpation and/or intraoperative ultrasonography to rule out tumor extension, en-bloc excision of a venous tumor thrombus	Complete tumor removal, en-bloc resection of the tumor thrombus together with the primary kidney tumor to avoid tumor tissue dissection and upstaging of the tumor	(43)
Lymph node	Sampling of hiliar, paracaval and paraaortic lymph nodes (and suspicious mesentery lymph nodes)	Local staging, sampling of more than seven lymph nodes	(34, 41)
Ipsilateral adrenal gland	Can be left *in situ* when easily separated from the tumor kidney and when without signs of tumor involvement	Incidence of tumor invasion into the gland in <5% of cases	(44)
Diaphragm	En-bloc resection in case of adherent tumor	To avoid spillage dissecting the tumor off the diaphragm	(30)
Liver	Extensive en-bloc resection or partial hepatectomy is not recommended in case of direct spread	Minimize secondary liver complications, no benefit shown for survival in case of extensive hepatic resection	(24, 45)
Bowel	Partial resection of intestine/colon	In case of tumor infiltration	
Peritoneum	Peritoneal exploration	Local staging, sign of tumor extension	
Contralateral kidney	Exploration	Only in case of a suggested contralateral kidney lesion or enlarged contralateral lymph nodes in pre-operative imaging, exploration should be done prior to tumor nephrectomy of the primarily involved kidney to adapt the surgical approach intraoperatively	(24, 38, 46)

Special (surgical) considerations have to be respected in case of large tumors, ruptured tumors, intravascular tumor extension, bilateral tumors, tumors in syndromic patients and tumors in a horseshoe kidney.

#### Large Tumors and Tumor Rupture

Preoperative tumor rupture or intraoperative tumor spill is of particular concern in WT as it creates a risk for local recurrence (24, 47). Spontaneous or traumatic tumor rupture is detected on preoperative imaging at a rate ranging between 3 and 23% (24, 48, 49). Intraoperatively, tumors larger than 12 cm in diameter are noted to be at risk for rupture (50). Patients treated according to the NWTS/COG protocol (i.e., no preoperative chemotherapy) have a risk of intraoperative tumor spillage of up to 10% (38, 50). An intraoperative spillage rate of 12% has been reported by SIOP in patients who had not received preoperative chemotherapy treatment (51). However, among SIOP-treated patients with neoadjuvant chemotherapy, the risk for intraoperative tumor spillage had decreased to around 3%. This is advocated as one of the major surgical advantages of the SIOP approach (51, 52). Once rupture of the tumor capsule is evident (pre- or intraoperatively), NWTS/COG stage I-II tumors are upgraded to stage III tumors. Upon preoperative surgical assessment, the NWTS/COG protocol provides the option for neoadjuvant chemotherapy when preoperative tumor rupture is evident, tumor spill is likely at surgery or the tumor deems unresectable without significant morbidity due to its size (24). The UMBRELLA SIOP-RTSG 2016 protocol also adjusts treatment to stage III when viable tumor cells are detected microscopically at the area of rupture (34). In addition to the upstaging of the tumor with intensified chemotherapy, whole abdominal irradiation for diffuse tumor spillage or an irradiation boost to the flank is applied in most cases (30, 53).

#### Intravascular Extension

In 4–10% of patients with WT, the tumor extends into the renal vein or inferior vena cava, whereas an extension into the right atrium or ventricle occurs in around 1–3% of cases (43, 54–57). A complete picture of a possible intravascular extension is essential for planning anesthesia and surgery. For example, an extensive tumor thrombus to the cardiac atrium may lead to cardiac deprivation and the need for cardiopulmonary bypass surgery for its resection (31, 56, 58). As the venous tumor extension is not always seen on preoperative imaging, intraoperative exploration by manual palpation and/or ultrasonography of the renal vein is mandatory. In general, excision of a tumor thrombus needs to be achieved in continuity, as dissection will upstage the tumor to stage III. The NWTS/COG protocol therefore recommends neoadjuvant treatment when intravascular tumor extends up to or above the hepatic veins (43, 59). It has been demonstrated that in 45–87% of patients with a tumor thrombus in their inferior vena cava, preoperative chemotherapy led to a size reduction improving surgical conditions (43, 60, 61). However, cardiac tumor extensions are often less responsive to neoadjuvant therapy, but in cases of good responds, cardiopulmonary bypass surgery can be avoided (43, 56).

#### Bilateral WT

About 6–7% of patients develop synchronous and <1% metachronous bilateral WT (15, 62). The prevalence is higher in patients with genetic predisposition syndromes carrying WT1 gene mutations and loss of imprinting (LOI) at 11p15 (15). As these genetic alterations are driving factors for early-disrupted embryologic differentiation of the kidney tissue, (multifocal) nephrogenic rests or nephroblastomatosis lesions are often seen in bilateral WT (63, 64). Conditions predisposing to metachronous bilateral WT are Denys-Drash syndrome, WAGR syndrome, Beckwith-Wiedemann syndrome, Fanconi anemia and familial WT. In these patients, up to 90% of WTs occur within the first 7 years of life necessitating close routine screening programs (15, 64).

Data from early studies revealed that patients with bilateral WT have a higher risk to suffer from end-stage renal disease (ESRD) within 20 years after treatment (cumulative incidence of 12% in bilateral WT vs. <1% in unilateral WT) (65). In patients with predisposing conditions, renal insufficiency rates are even higher (e.g., 83% in Denys-Drash syndrome and 43% in WAGR syndrome) (66, 67). In cases of synchronous or potentially metachronous bilateral WT, renal tissue can be preserved by NSS. Best conditions for successful NSS are low volume tumors in the kidney's periphery (67, 68). Neoadjuvant chemotherapy to confine the tumor enabling surgical resectability is therefore part of all treatment protocols. In a selected group of patients with bilateral WT, bilateral nephrectomy and kidney transplantation may be considered as a treatment option to eliminate the risk of initial or metachronous WT development (68, 69). This subgroup includes patients with Denys-Drash syndrome in whom prophylactic or secondary bilateral nephrectomy (in case of ESRD) is an acceptable procedure (64, 70, 71). In these patients, kidney transplantation is usually performed after 1–2 years of disease-free survival (64, 67).

#### Horseshoe and Solitary Kidneys

Wilms tumor in a horseshoe kidney is an exceptionally unique situation. There is a historic collection of 41 patients reported from the NWTS/COG (72). Surgical aims are the same as for unilateral WT with complete tumor nephroureteroectomy and lymph node sampling. However, some surgeons advocate NSS (73). The treatment of WT in solitary kidneys is guided by the same principles as for bilateral WT (30).

## Recent Technical Innovations

### Nephron-Sparing Surgery

Performance of NSS is a necessity in children presenting with bilateral WT (74). In unilateral WT, NSS must be considered in syndromic patients with an increased risk of metachronous development of (contralateral) WT, in patients with a solitary kidney or an afunctional contralateral kidney as well as in patients who are at risk of kidney failure (46). In non-syndromic unilateral WT, NSS is not a standard procedure (75). Potential benefits are prevention of functional renal impairment and ESRD, whereas some long-term follow-up data suggest good renal function even after unilateral radical nephrectomy (RN) for WT (65, 76–79). However, higher rates of postoperative stage III WTs due to positive resection margins have been reported (75). This required conversion to RN and/or additional radiation therapy. Still, EFS and OS were comparable to patients with RN (75, 80). If NSS is considered in unilateral WT, careful patient selection should be performed preoperatively (80). Davidoff et al. (81) prospectively analyzed patients on NSS feasibility according to radiological imaging and concluded that 8% of patients could be treated with NSS. The SIOP-RTSG surgical panel formulated a list of preconditions under which NSS for unilateral WT may be performed (i.e., tumor restricted to one pole, tumor volume <300 ml, no tumor rupture, no tumor in renal pelvis, no continuous organ invasion, no venous tumor thrombus, functioning kidney remnant after NSS) (30, 82).

Nephron-sparing surgery can be carried out by partial nephrectomy (i.e., tumor resection with a rim of normal renal tissue) or enucleation of the tumor (i.e., tumor resection without a rim of normal renal tissue) (30). At present, there are no available data comparing both methods. Method selection is highly dependent on tumor localization, size and the presence of multifocal lesions.

The main aim of NSS is to preserve as much healthy kidney tissue as possible. Preoperative chemotherapy may contribute to the preservation of renal tissue. The COG-AREN0534 reviewed 34 patients with predisposing conditions to bilateral WT presenting with unilateral tumors. This study showed that partial nephrectomy was feasible in 65% of patients after neoadjuvant chemotherapy, avoiding tumor nephrectomy and sparing renal tissue without compromising EFS and OS (46).

To assure a bloodless dissection and good visibility when dissecting through the kidney's parenchyma, intermittent clamping of the renal vasculature may be helpful. Some surgeons have advocated adopting operative techniques from adult kidney cancer surgery such as inducing renal hypothermia (e.g., application of ice water into the kidney bed) in the attempt to maximize the preservation of kidney tissue (62, 83, 84). As this is not yet a standard approach, future studies will need to address possible outcome benefits of this technique.

Another surgical adjunct is the use of intraoperative ultrasound in NSS. For example, ultrasound-guided mapping of the tumor nodules can aid to optimize surgical dissection lines, thus preserving healthy kidney tissue. Still, its use has to be critically reviewed as it does not guarantee tumor-free resection margins (83).

### Minimally Invasive Approaches

Laparoscopic RN and laparoscopic-assisted partial nephrectomy is becoming more common for the treatment of WT as comparable outcomes have been reported. Nevertheless, the outcome analysis may be biased by the fact that laparoscopically resected tumors have lower stages of disease (75, 85–90). Additionally, robotic-assisted laparoscopy (RAL) is a developing field in pediatric surgical oncology (91–94). However, the experience of RAL in WT surgery is yet limited and its indications need to be carefully discussed in tumor and surgical reference boards (82, 94). In general, minimally invasive tumor resection is limited to tumors confined to the kidney with good exposure of the hilar vessels (85, 95). Large tumors may not leave enough operating space for laparoscopy. Therefore, patient selection is of the utmost importance (95, 96). In the UMBRELLA SIOP-RTSG 2016 protocol, the following prerequisites for laparoscopic RN including RAL have been proposed: small, central tumors with a rim of non-malignant renal tissue, extraction of the specimen in a bag without morcellation, no venous tumor thrombus, no continuous organ infiltration, no extension of the tumor beyond the ipsilateral boarder of the spine, no imminent tumor rupture (i.e., in case of no response to chemotherapy) and feasibility of lymph node sampling as well as the operating surgeon is expected to be experienced in minimally invasive nephrectomy (30, 82). In all instances, performance of minimally invasive surgery must adhere to the same general surgical principles of WT treatment such as complete lymph node sampling and thoroughness of resection (30).

## Advances in Tumor Imaging Technology

Diffusion-weighted imaging (DWI) MRI technique has added a new quality to the available imaging modalities for WT. By defining the whole-tumor apparent diffusion coefficient, it provides information on the “cell density” of a tumor lesion, assisting the radiologist in defining necrotic from viable lesions after chemotherapy and small lesions in nephroblastomatosis (97–99). Imaging research studies aim at defining histological subtypes of WTs by DWI for radiologic assistance in risk stratification (100–102).

Detecting hiliar and retroperitoneal metastatic lymph node disease is critically important in WT (103, 104). Lymph node metastasis can be occult and may not necessarily be apparent by lymph node enlargement during the operation. To improve lymph node sampling in WT, first feasibility studies have been conducted to establish the concept of intraoperative sentinel lymph node detection. Two techniques have been described in the attempt to delineate the lymphatic drainage of the involved kidney: injection of a radioactive tracer (e.g., technetium-99 m phytate detected with an intraoperative gamma probe) or a fluorescence dye (e.g., indocyanine green visible under near-infra red laparoscopy) (103, 104). In this recently published study, including unilateral WTs with indication for tumor nephrectomy, sentinel lymph nodes were most frequently detected in the aorto-caval space after injection of a radioactive tracer into the normal kidney tissue adjacent to the WT (104). Further systematic studies will be needed to standardize and verify the advantages of this technique.

Surgical research on fluorescence-guided kidney surgery has also evolved. Surgical procedures delineating perfusion of the kidney including separation of the upper and lower pole, visualizing renal masses (cysts) or mapping lymphatic drainage (e.g., in lymphatic sparing varicocelectomy) have been successfully executed (105, 106). These new surgical imaging techniques may eventually have the potential to transform the surgical approach in WT treatment.

## Prognostic Biomarkers

The number of newly identified biomarkers in WT is growing constantly. To date, more than 30 (epi-)genetic and protein biomarkers have been suggested (107). Most of these biomarkers are closely linked to tumorigenesis and predisposition of WT. Some may eventually play a role in targeted therapy. One such candidate is an antagonist of the Wnt/beta-catenin pathway, tegavivint (BC2059), which is currently under investigation by the COG for its antitumor activity in recurrent and refractory pediatric solid tumors including WT. Currently, patients with WT-associated CTNNB1 oncogene mutations leading to Wnt/beta-catenin pathway activation can be included in this study (https://clinicaltrials.gov/ct2/show/NCT04851119) (108).

So far, only one genetic biomarker, i.e., LOH at chromosomes 1p/16q, has been integrated as a decision-making factor into the current COG treatment protocol. In the presence of LOH at chromosomes 1p/16q, patients with stage I and II favorable WT histology will be upstaged from low to standard risk receiving regimen DD-4A (i.e., vincristine, dactinomycin and doxorubicine for 24 weeks) instead of regimen EE-4A (i.e., vincristine and actinomycin D for 16 weeks) after nephrectomy. The previous NWTS-5 showed a significantly improved EFS and OS in patients with intensified therapy (27). Intensified chemotherapy is also given to patients with LOH at chromosomes 1p/16q and lung metastasis in the NWTS/COG protocol. Although it serves as a sensitive marker for risk stratification, LOH at chromosomes 1p/16q is only applicable in a small subset of patients and presently it does not offer possible treatment targets.

With 30% of favorable histology WT cases, one of the most prevalent outcome predictors is gain of function (GOF) at chromosome 1q. It is associated with significantly poorer EFS and OS, as reported by the NWTS/COG and SIOP study groups, and is currently under reinvestigation in the UMBRELLA SIOP-RTSG protocol for its prognostic value (30, 109, 110).

In general, WT is considered an embryonal tumor consisting of primordial renal cells disrupted to mature into differentiated kidney tissue. Variable proportions of blastemal (i.e., renal stem cells), epithelial and stromal cells have great influence on tumor behavior and outcome. During kidney development, organogenesis passes the stage of nephrogenic differentiation. This stage of development can persist as intra- or perilobar nephrogenic rests throughout the first few years of life and is a very likely origin for WT tumorigenesis. One important factor in driving embryonal renal differentiation is the transcription factor WT1 on chromosome 11p13. Its mutation is linked to the development of WT. WT1 mutations are reported in 10–20% of sporadic WT cases (111). Together with mutations in the CTNNB1 and AMER1 (WTX) tumor suppressor genes, it unfolds its tumorigenic role by upregulation of the Wnt/beta-catenin pathway (112). As germline alterations, WT1 mutations are common in syndromic predisposition syndromes (e.g., Denys-Drash syndrome and WAGR syndrome), bilateral WT and WT with synchronous nephrogenic rests (5, 113, 114). Another transcription factor that plays a role in bilateral as well as in predisposing syndromes is WT2 on chromosome 11p15. Mutations in this gene are much more abundant (i.e., 70% of cases) with 40% in sporadic cases and 30% in Beckwith-Wiedemann syndrome (115).

In recent research, mutations have been identified in microRNA (miRNA) processing genes in about 20% of patients with WT (116, 117). These alterations in miRNA processing are thought to conserve the embryonal stage of kidney development (116, 118). However, data concerning their influence of clinical behavior in WT have not yet been specifically reported.

When analyzing molecular markers in tumor tissues, it is important to consider that tumors consist of miscellaneous tissue parts containing different levels of marker expression. This has been shown for GOF at chromosome 1q, leaving a general uncertainty in biopsies (109, 119).

In the majority of cases, biomarkers identify WTs with treatment resistance and poor prognosis. Table 4 provides an overview of transcription factors, oncogenes and tumor suppressor genes directing at multiple different pathways potentially involved in tumorigenesis of WT.

**Table 4:** List of selected biomarkers with potential relevance for WT prognosis and/or tumorigenesis.

**Biomarkers, Gene**	**Incidence**	**Relevant findings**	**Reference**
CTNNB1	15%	– Stromal predominant histology – Upregulation of the Wnt/beta-catenin pathway – Patients with CTNNB1 mutations leading to upregulation of the Wnt/beta-catenin pathway are currently included in phase II trials as a possible treatment target for tegavivint (BC2059)	(10, 108, 111)
LOH 1p/16q	5.0% in WT with favorable histology and 9.4% in relapsed WT	– NWTS-5: LOH 1p/16q predicted inferior 4-year EFS and OS in stage III and IV tumors	(11, 107)
LOH 1p	12%	– NWTS-5: Significantly increased rate of relapse and decreased OS independent of tumor stage and histology – Predicting WT relapse (RR 2.93)	(11, 107)
LOH 16q	17%	– NWTS-5: Significantly increased rate of relapse and decreased OS independent of tumor stage and histology – Predicting WT relapse (RR 1.95)	(11, 107)
GOF 1q	30.0% overall and 18.3% in stage IV WT	– No histologic pre-dominance – NWTS-5: Inferior 4-year EFS and OS in stage I, III and IV tumors – Predicting relapse (RR 2.86) unrelated to tumor stage	(107, 109, 110)
WT1 (chr. 11p13)	10–20%	– Predominant stromal histology – Simultaneous presence of intralobar nephrogenic rests – WT1 mutations and LOH 11p15 associated with relapse – Germline mutations in Denys-Drash syndrome and WAGR syndrome – 90% of patients with Denys-Drash syndrome and 50% with WAGR syndrome develop WT	(5, 111, 112, 114, 120)
WT2 (chr. 11p15)	70%	– Germline mutation in Beckwith-Wiedemann syndrome – 4–5% of patients with Beckwith-Wiedemann syndrome develop WT – IGF2 upregulation	(115)
LOH 11p15		– IGF2 upregulation – Increased risk of recurrence – Described in anaplastic WT, relapse, and fatal cases	(107)
LOI 11p15	30–50%	– Leading to H19 and IGF2 activation and unrestrained cell growth – Predicting relapse in low-risk WT treated with surgery alone	(120, 121)
WTX (AMER1)	15–20%	– Upregulation of the Wnt/beta-catenin pathway – Combined with epigenetic 11p15 alterations	(111, 122)
miRNA processing genes	20%	– Mutations in miRNA processing genes including DROSHA (80% of miRNA mutations in WT), DGCR8 and DIS3L2 (Perlman syndrome)	(116–118)
MLLT1	4%	– High prevalence of intralobar nephrogenic rests	(123)
MYCN	<10%	– Described in treatment resistance, relapse, and fatal cases – Detected at higher proportion of pre-treated anaplastic WT (>30%), potential marker for treatment resistance	(124)
LOH 11q		– Higher detection in mixed and diffuse anaplastic WT – Associated with recurrence and fatal cases	(124)
SIX1 and SIX2	5–10%	– Blastemal predominant histology	
TRIM28	5%	– Mature epithelial histology predominant – Excellent prognosis – Frequent in bilateral and familial cases	(107, 125, 126)
TP53 (chr. 17p13)	5%	– 75% in diffuse anaplastic tumors – Found primarily in advanced tumor stages – Significantly poorer outcome in stage III and IV anaplastic tumors – Linked to increased recurrence	(105, 106)
CTR9	Described in four families and sporadic cases	– Non-syndromic WT predisposition	(127)

*GOF, gain of function; LOH, loss of heterozygosity; LOI, loss of imprinting; WT, Wilms tumor*.

## Future Directions

The concept of “personalized medicine” has already improved the treatment of children with WT. The future trend will increasingly be driven by two principles: (1) de-escalation of therapy with the aim to define the appropriate level of treatment to achieve the best outcome while minimizing secondary side effects, and (2) identifying tumor-related and personal risk factors justifying escalation of therapy. To address these principles, the two large WT consortia of the COG and SIOP have designed new prospective studies (i.e., AREN03B2 umbrella study within the COG registry “*Project: Every Child”* and UMBRELLA SIOP-RTSG protocol, respectively) to collect and overlay clinical and biological data on a profound scale. Within these registries, both groups have established systematic biospecimen banks with tumor and blood samples available for prospective and retrospective analysis. Due to the fact that many prognostic (bio-)markers, such as blastemal histology or LOH at chromosomes 1p/16q, are only identified in a small subset of patients, treatment centers worldwide are encouraged to enroll patients (30).

Molecular research has identified a multitude of genetic and protein biomarkers for WT that may eventually assign patients into more specific risk groups. Among the most prevalent and promising prognostic markers for adverse outcome is GOF at chromosome 1q, which is currently under investigation as part of the UMBRELLA SIOP-RTSG protocol. Other markers may potentially serve as therapeutic targets. Patients with recurrent or refractory WT and alteration of the Wnt/beta-catenin pathway can be included in a therapeutic phase I/II application study of tegavivint (BC2059). The study is open for a number of different pediatric solid tumors with refractory treatment response and mutational activation of the Wnt/beta-catenin pathway.

Genomic sequencing programs such as TARGET (Therapeutically Applicable Research to Generate Effective Treatments) in the United States and FACT (Factors Associated with Childhood Tumors) in the United Kingdom have accelerated the discovery of inherited and acquired factors possibly responsible for the development of WT (128–130). The growing knowledge on tumor biology and genetics will increasingly influence the decision-making process, and contribute to the general understanding of tumorigenesis of WT.

Another important field of research with respect to WT biomarkers is the identification of tumor DNA in blood or urine samples. In patients enrolled in SIOP and UKCCSG/CCLG (United Kingdom Children's Cancer Study Group/Children's Cancer and Leukemia Group), a 5–12% rate of misdiagnosis has been reported in patients without biopsy or primary surgery (131). In the near future, so-called “liquid biopsies” may aid in establishing WT diagnosis and screening patients in follow-up programs for recurrent WT (131–133).

Recent studies have focused on the global epidemiology of WT, offering a broad picture of who is at risk for the development of WT not only by world region, ethnic background, gender and age, but also by socioeconomic status and health care accessibility (1, 3, 134). The highest WT incidence rates have been reported in North America in children of African-American descent, whereas the lowest was seen in children in East Asia (2). Differences in genetics have also been reported by ethnicity and world region. For instance, the genetic alteration of the IGF-2 gene locus playing the driving role in overgrowth syndromes and WT was less frequently seen in children with WT from Japan in comparison to Caucasian children (2, 135, 136). Future research will need to overlay epidemiology with genetic data in larger patient cohorts to identify populations at risk for WT development, treatment resistance or worse outcome.

Centralized review of WT imaging and pathology as well as treatment guidance has more and more been integrated in current treatment protocols. In previous studies, discrepancies in institutional and central histopathology interpretation have been reported in 20–50% of cases (9, 35). Ultimately, centralized data review may lead to more coherent data sets.

Despite significant progress in molecular biology, research defining biomarkers and identification of new treatment targets for WT, technical advances in imaging, surgery and radiation therapy will evenly be important. In the future, artificial intelligence algorithms of tumor imaging and 3-D reconstructions of WT may assist the surgeon in defining surgical resection lines, thus improving surgical outcome particularly in cases of NSS (106, 137). In addition, advances in the application of radiotherapy such as intensity-modulated radiation therapy sparing non-tumorous tissue and limiting radiation-associated toxicity will be beneficial for the WT patients (138, 139).

## Author Contributions

T-MT, YB, KB, UR, HF, and FF performed the literature search for the work. T-MT and YB outlined and wrote the initial manuscript draft. KB, UR, HF, and FF critically revised the initial manuscript draft for important intellectual content. All authors approved the final version to be published and agree to be accountable for all aspects of the work in ensuring that questions related to the accuracy or integrity of any part of the work are appropriately investigated and resolved.

## Funding

This work was supported by the German Research Foundation and the Institutional Open Access Fund of the Goethe University Frankfurt within the program of Open Access Publishing.

## Conflict of Interest

The authors declare that the research was conducted in the absence of any commercial or financial relationships that could be construed as a potential conflict of interest.

## Publisher's Note

All claims expressed in this article are solely those of the authors and do not necessarily represent those of their affiliated organizations, or those of the publisher, the editors and the reviewers. Any product that may be evaluated in this article, or claim that may be made by its manufacturer, is not guaranteed or endorsed by the publisher.

## References

[1] Cunningham ME Klug TD Nuchtern JG Chintagumpala MM Venkatramani R Lubega J. Global disparities in Wilms tumor. J Surg Res. (2020) 247:34–51. 10.1016/j.jss.2019.10.044 31810638

[2] Nakata K Colombet M Stiller CA Pritchard-Jones K Steliarova-Foucher E IICC-3 Contributors. Incidence of childhood renal tumors: an international population-based study. Int J Cancer. (2020) 147:3313–27. 10.1002/ijc.33147 32902866 PMC7689773

[3] Steliarova-Foucher E Colombet M Ries LAG Moreno F Dolya A Bray F. International incidence of childhood cancer, 2001–10: a population-based registry study. Lancet Oncol. (2017) 18:719–31. 10.1016/S1470-2045(17)30186-9 28410997 PMC5461370

[4] Pastore G Znaor A Spreafico F Graf N Pritchard-Jones K Steliarova-Foucher E. Malignant renal tumors incidence and survival in European children (1978–1997): report from the automated childhood cancer information system project. Eur J Cancer. (2006) 42:2103–14. 10.1016/j.ejca.2006.05.010 16919774

[5] Breslow NE Beckwith JB Perlman EJ Reeve AE. Age distributions, birth weights, nephrogenic rests, and heterogeneity in the pathogenesis of Wilms tumor. Pediatr Blood Cancer. (2006) 47:260–7. 10.1002/pbc.20891 16700047 PMC1543666

[6] Green DM. The evolution of treatment for Wilms tumor. J Pediatr Surg. (2013) 48:14–9. 10.1016/j.jpedsurg.2012.10.012 23331787

[7] Raffensperger J. Max Wilms and his tumor. J Pediatr Surg. (2015) 50:356–9. 10.1016/j.jpedsurg.2014.10.054 25638637

[8] Wright JC. Update in cancer chemotherapy: genitourinary tract cancer, part 2: Wilms' tumor and bladder cancer. J Natl Med Assoc. (1988) 80:169–81. 2853770 PMC2625724

[9] Dome JS Mullen EA Dix DB Gratias EJ Ehrlich PF Daw NC. Impact of the first generation of children's oncology group clinical trials on clinical practice for Wilms tumor. J Natl Compr Canc Netw. (2021) 19:978–85. 10.6004/jnccn.2021.7070 34416705 PMC8805512

[10] Spreafico F Fernandez CV Brok J Nakata K Vijanic G Geller J. Wilms tumor. Nat Rev Dis Primers. (2021) 7:75. 10.1038/s41572-021-00308-8 34650095

[11] Grundy PE Breslow NE Li S Perlman E Beckwith JB Ritchey ML. Loss of heterozygosity for chromosomes 1p and 16q is an adverse prognostic factor in favorable-histology Wilms tumor: a report from the national Wilms tumor study group. J Clin Oncol. (2005) 23:7312–21. 10.1200/JCO.2005.01.2799 16129848

[12] Grundy PE Telzerow PE Breslow N Moksness J Huff V Paterson MC. Loss of heterozygosity for chromosomes 16q and 1p in Wilms' tumors predicts an adverse outcome. Cancer Res. (1994) 54:2331–3. 8162576

[13] Brok J Lopez-Yurda M Tinteren HV Treger TD Furtwängler R Graf N. Relapse of Wilms' tumor and detection methods: a retrospective analysis of the 2001 renal tumor study group-international society of pediatric oncology Wilms' tumor protocol database. Lancet Oncol. (2018) 19:1072–81. 10.1016/S1470-2045(18)30293-6 29960848

[14] Daw NC Chi YY Kalapurakal JA Kim Y Hoffer FA Geller JI. Activity of vincristine and irinotecan in diffuse anaplastic Wilms tumor and therapy outcomes of stage II to IV disease: results of the children's oncology group AREN0321 study. J Clin Oncol. (2020) 38:1558–68. 10.1200/JCO.19.01265 32134700 PMC7213587

[15] Charlton J Irtan S Bergeron C Pritchard-Jones K. Bilateral Wilms tumor: a review of clinical and molecular features. Expert Rev Mol Med. (2017) 19:e8. 10.1017/erm.2017.8 28716159 PMC5687181

[16] Ehrlich P Chi YY Chintagumpala MM Hoffer FA Perlman EJ Kalapurakal JA. Results of the first prospective multi-institutional treatment study in children with bilateral Wilms tumor (AREN0534): a report from the children's oncology group. Ann Surg. (2017) 266:470–8. 10.1097/SLA.0000000000002356 28795993 PMC5629006

[17] Verschuur A Van Tinteren H Graf N Bergeron C Sandstedt B de Kraker J. Treatment of pulmonary metastases in children with stage IV nephroblastoma with risk-based use of pulmonary radiotherapy. J Clin Oncol. (2012) 30:3533–9. 10.1200/JCO.2011.35.8747 22927531

[18] Grundy PE Green DM Dirks AC Berendt A Breslow NE Anderson JR. Clinical significance of pulmonary nodules detected by CT and Not CXR in patients treated for favorable histology Wilms tumor on national Wilms tumor studies-4 and−5: a report from the children's oncology group. Pediatr Blood Cancer. (2012) 59:631–5. 10.1002/pbc.24123 22422736 PMC3397278

[19] Warmann SW Furtwangler R Blumenstock G Armeanu S Nourkami N Leuschner I. Tumor biology influences the prognosis of nephroblastoma patients with primary pulmonary metastases: results from SIOP 93-01/GPOH and SIOP 2001/GPOH. Ann Surg. (2011) 254:155–62. 10.1097/SLA.0b013e318222015e 21670612

[20] Termuhlen AM Tersak JM Liu Q Yasui Y Stovall M Weathers R. Twenty-five year follow-up of childhood Wilms tumor: a report from the childhood cancer survivor study. Pediatr Blood Cancer. (2011) 57:1210–6. 10.1002/pbc.23090 21384541 PMC4634648

[21] Wong KF Reulen RC Winter DL Guha J Fidler MM Kelly J. Risk of adverse health and social outcomes up to 50 years after Wilms tumor: the British childhood cancer survivor study. J Clin Oncol. (2016) 34:1772–9. 10.1200/JCO.2015.64.4344 27022116

[22] Golden CB Feusner JH. Malignant abdominal masses in children: quick guide to evaluation and diagnosis. Pediatr Clin North Am. (2002) 49:1369–92. 10.1016/S0031-3955(02)00098-6 12580370

[23] Irtan S Ehrlich PF Pritchard-Jones K. Wilms tumor: state-of-the-art update, 2016. Semin Pediatr Surg. (2016) 25:250–6. 10.1053/j.sempedsurg.2016.09.003 27955727

[24] Shamberger RC Guthrie KA Ritchey ML Haase GM Takashima J Beckwith JB. Surgery-related factors and local recurrence of Wilms tumor in national Wilms tumor study 4. Ann Surg. (1999) 229:292–7. 10.1097/00000658-199902000-00019 10024113 PMC1191644

[25] Irtan S Van Tinteren H Graf N van den Heuvel-Eibrink MM Heij H Bergeron C. Evaluation of needle biopsy as a potential risk factor for local recurrence of Wilms tumor in the SIOP WT 2001 trial. Eur J Cancer. (2019) 116:13–20. 10.1016/j.ejca.2019.04.027 31163337

[26] Jackson TJ Williams RD Brok J Chowdhury T Ronghe M Powis M. The diagnostic accuracy and clinical utility of pediatric renal tumor biopsy: report of the UK experience in the SIOP UK WT 2001 trial. Pediatr Blood Cancer. (2019) 66:e27627. 10.1002/pbc.27627 30761727 PMC6522371

[27] Dix DB Seibel NL Chi YY Khanna G Gratias E Anderson JR. Treatment of stage IV favorable histology Wilms tumor with lung metastases: a report from the children's oncology group AREN0533 study. J Clin Oncol. (2018) 36:1564–70. 10.1200/JCO.2017.77.1931 29659330 PMC6075846

[28] Green DM Fernbach DJ Norkool P Kollia G D'Angio GJ. The treatment of Wilms' tumor patients with pulmonary metastases detected only with computed tomography: a report from the national Wilms' tumor study. J Clin Oncol. (1991) 9:1776–81. 10.1200/JCO.1991.9.10.1776 1655987

[29] Smets AM van Tinteren H Bergeron C De Camargo B Graf N Pritchard-Jones K. The contribution of chest CT-scan at diagnosis in children with unilateral Wilms' tumor. Results of the SIOP 2001 study. Eur J Cancer. (2012) 48:1060–5. 10.1016/j.ejca.2011.05.025 21703848

[30] van den Heuvel-Eibrink MM Hol JA Pritchard-Jones K van Tinteren H Furtwängler R Verschuur AC. Position paper: rationale for the treatment of Wilms tumor in the UMBRELLA SIOP-RTSG 2016 protocol. Nat Rev Urol. (2017) 14:743–52. 10.1038/nrurol.2017.163 29089605

[31] Fanelli MCA Guilhen JCS Duarte AAB Marques de Souza FK Dos Santos Cypriano M Caran EMM. Management of pediatric tumors with vascular extension. Front Pediatr. (2021) 9:753232. 10.3389/fped.2021.753232 35059362 PMC8764352

[32] Khanna G Rosen N Anderson JR Ehrlich PF Dome JS Gow KW. Evaluation of diagnostic performance of CT for detection of tumor thrombus in children with Wilms tumor: a report from the children's oncology group. Pediatr Blood Cancer. (2012) 58:551–5. 10.1002/pbc.23222 21674767 PMC3175263

[33] Brisse HJ Smets AM Kaste SC Owens CM. Imaging in unilateral Wilms tumor. Pediatr Radiol. (2008) 38:18–29. 10.1007/s00247-007-0677-9 18038168

[34] Vujanic GM Gessler M Ooms A Collini P Coulomb-l'Hermine A D'Hooghe E. The UMBRELLA SIOP-RTSG 2016 Wilms tumor pathology and molecular biology protocol. Nat Rev Urol. (2018) 15:693–701. 10.1038/s41585-018-0100-3 30310143 PMC7136175

[35] Perlman EJ. Pediatric renal tumors: practical updates for the pathologist. Pediatr Dev Pathol. (2005) 8:320–38. 10.1007/s10024-005-1156-7 16010493

[36] Hamilton TE Barnhart D Gow K Ferrer F Kandel J Glick R. Inter-rater reliability of surgical reviews for AREN03B2: a COG renal tumor committee study. J Pediatr Surg. (2014) 49:154–8. 10.1016/j.jpedsurg.2013.09.047 24439601 PMC4076163

[37] Lopes RI Lorenzo A. Recent advances in the management of Wilms' tumor. F1000Res. (2017) 6:670. 10.12688/f1000research.10760.1 28620463 PMC5461897

[38] Ehrlich PF Ritchey ML Hamilton TE Haase GM Ou S Breslow N. Quality assessment for Wilms' tumor: a report from the national Wilms' tumor study-5. J Pediatr Surg. (2005) 40:208–13. 10.1016/j.jpedsurg.2004.09.044 15868587

[39] Kieran K Anderson JR Dome JS Ehrlich PF Ritchey ML Shamberger RC. Lymph node involvement in Wilms tumor: results from national Wilms tumor studies 4 and 5. J Pediatr Surg. (2012) 47:700–6. 10.1016/j.jpedsurg.2011.08.017 22498384 PMC3976547

[40] Zhuge Y Cheung MC Yang R Koniaris LG Neville HL Sola JE. Improved survival with lymph node sampling in Wilms tumor. J Surg Res. (2011) 167:e199–203. 10.1016/j.jss.2010.12.026 21324394

[41] Ehrlich PF Anderson JR Ritchey ML Dome JS Green DM Grundy PE. Clinicopathologic findings predictive of relapse in children with stage III favorable-histology Wilms tumor. J Clin Oncol. (2013) 31:1196–201. 10.1200/JCO.2011.41.1165 23382471 PMC3595426

[42] Ritchey ML Kelalis PP Breslow N Offord KP Shochat SJ D'Angio GJ. Intracaval and atrial involvement with nephroblastoma: review of national Wilms tumor study-3. J Urol. (1988) 140:1113–8. 10.1016/S0022-5347(17)41975-6 2846897

[43] Shamberger RC Ritchey ML Haase GM Bergemann TL Loechelt-Yoshioka T Breslow NE. Intravascular extension of Wilms tumor. Ann Surg. (2001) 234:116–21. 10.1097/00000658-200107000-00017 11420491 PMC1421956

[44] Kieran K Anderson JR Dome JS Ehrlich PF Ritchey ML Shamberger RC. Is adrenalectomy necessary during unilateral nephrectomy for Wilms Tumor? A report from the children's oncology group. J Pediatr Surg. (2013) 48:1598–603. 10.1016/j.jpedsurg.2013.04.019 23895979 PMC5652039

[45] Ehrlich PF Ferrer FA Ritchey ML Anderson JR Green DM Grundy PE. Hepatic metastasis at diagnosis in patients with Wilms tumor is not an independent adverse prognostic factor for stage IV Wilms tumor: a report from the children's oncology group/national Wilms tumor study group. Ann Surg. (2009) 250:642–8. 10.1097/SLA.0b013e3181b76f20 19730241 PMC3302661

[46] Ehrlich PF Chi YY Chintagumpala MM Hoffer FA Perlman EJ Kalapurakal JA. Results of treatment for patients with multicentric or bilaterally pre-disposed unilateral Wilms tumor (AREN0534): a report from the children's oncology group. Cancer. (2020) 126:3516–25. 10.1002/cncr.32958 32459384 PMC7769115

[47] Khanna G Naranjo A Hoffer F Mullen E Geller J Gratias EJ. Detection of pre-operative Wilms tumor rupture with CT: a report from the children's oncology group. Radiology. (2013) 266:610–7. 10.1148/radiol.12120670 23192775 PMC3558872

[48] Brisse HJ Schleiermacher G Sarnacki S Helfre S Philippe-Chomette P Boccon-Gibod L. Pre-operative Wilms tumor rupture: a retrospective study of 57 patients. Cancer. (2008) 113:202–13. 10.1002/cncr.23535 18457331

[49] Zhang Y Song HC Yang YF Sun N Zhang WP Huang CR. Pre-operative Wilms tumor rupture in children. Int Urol Nephrol. (2021) 53:619–25. 10.1007/s11255-020-02706-5 33245535

[50] Gow KW Barnhart DC Hamilton TE Kandel JJ Chen MK Ferrer FA. Primary nephrectomy and intraoperative tumor spill: report from the children's oncology group (COG) renal tumors committee. J Pediatr Surg. (2013) 48:34–8. 10.1016/j.jpedsurg.2012.10.015 23331790 PMC4556229

[51] Fuchs J Kienecker K Furtwängler R Warmann SW Bürger D Thürhoff JW. Surgical aspects in the treatment of patients with unilateral Wilms tumor: a report from the SIOP 93-01/German society of pediatric oncology and hematology. Ann Surg. (2009) 249:666–71. 10.1097/SLA.0b013e31819ed92b 19300220

[52] Graf N Tournade MF de Kraker J. The role of pre-operative chemotherapy in the management of Wilms' tumor. The SIOP studies. International society of pediatric oncology. Urol Clin North Am. (2000) 27:443–54. 10.1016/S0094-0143(05)70092-6 10985144

[53] Kalapurakal JA Li SM Breslow NE Beckwith JB Ritchey ML Shamberger RC. Intraoperative spillage of favorable histology Wilms tumor cells: influence of irradiation and chemotherapy regimens on abdominal recurrence. A report from the national Wilms tumor study group. Int J Radiat Oncol Biol Phys. (2010) 76:201–6. 10.1016/j.ijrobp.2009.01.046 19395185 PMC2843421

[54] Elayadi M Hammad M Sallam K Ahmed G Ahmed S Ibrahim A. Management and outcome of pediatric Wilms tumor with malignant inferior Vena cava thrombus: largest cohort of single-center experience. Int J Clin Oncol. (2020) 25:1425–31. 10.1007/s10147-020-01667-0 32249334

[55] Qureshi SS Bhagat M Smriti V Murli D Baheti A Yadav S. Intravascular extension of Wilms tumor: characteristics of tumor thrombus and their impact on outcomes. J Pediatr Urol. (2021) 17:69.e1–69.e8. 10.1016/j.jpurol.2020.10.003 33087302

[56] Cox SG Davidson A Thomas J Brooks A Hewitson J Numanoglu A. Surgical management and outcomes of 12 cases of Wilms tumor with intracardiac extension from a single center. Pediatr Surg Int. (2018) 34:227–35. 10.1007/s00383-017-4197-x 29022081

[57] Dzhuma K Powis M Vujanic G Watson T Olsen O Shelmerdine S. Surgical management, staging, and outcomes of Wilms tumors with intravascular extension: results of the IMPORT study. J Pediatr Surg. (2022) 57:572–8. 10.1016/j.jpedsurg.2021.08.023 34565577

[58] Abdullah Y Karpelowsky J Davidson A Thomas J Brooks A Hewitson J. Management of nine cases of Wilms' tumor with intracardiac extension—a single center experience. J Pediatr Surg. (2013) 48:394–9. 10.1016/j.jpedsurg.2012.11.024 23414872

[59] Kieran K Ehrlich PF. Current surgical standards of care in Wilms tumor. Urol Oncol. (2016) 34:13–23. 10.1016/j.urolonc.2015.05.029 26122713

[60] Xu S Sun N Zhang WP Song HC Huang CR. Management of Wilms tumor with intravenous thrombus in children: a single center experience. World J Pediatr. (2019) 15:476–82. 10.1007/s12519-019-00272-0 31161446 PMC6785647

[61] Hadley GP Sheik-Gafoor MH Buckels NJ. The management of nephroblastoma with cavo-atrial disease at presentation: experience from a developing country. Pediatr Surg Int. (2010) 26:1169–72. 10.1007/s00383-010-2667-5 20697900

[62] Millar AJ Davidson A Rode H Numanoglu A Hartley PS Daubenton JD. Bilateral Wilms' tumors: a single-center experience with 19 cases. J Pediatr Surg. (2005) 40:1289–94. 10.1016/j.jpedsurg.2005.05.013 16080934

[63] Coorens THH Treger TD Al-Saadi R Moore L Tran MGB Mitchell TJ. Embryonal pre-cursors of Wilms tumor. Science. (2019) 366:1247–51. 10.1126/science.aax1323 31806814 PMC6914378

[64] Liu EK Suson KD. Syndromic Wilms tumor: a review of pre-disposing conditions, surveillance and treatment. Transl Androl Urol. (2020) 9:2370–81. 10.21037/tau.2020.03.27 33209710 PMC7658145

[65] Breslow NE Collins AJ Ritchey ML Grigoriev YA Peterson SM Green DM. End stage renal disease in patients with Wilms tumor: results from the national Wilms tumor study group and the United States renal data system. J Urol. (2005) 174:1972–5. 10.1097/01.ju.0000176800.00994.3a 16217371 PMC1483840

[66] Lange J Peterson SM Takashima JR Grigoriev Y Ritchey ML Shamberger RC. Risk factors for end stage renal disease in non-WT1-syndromic Wilms tumor. J Urol. (2011) 186:378–86. 10.1016/j.juro.2011.03.110 21683387 PMC3133859

[67] Cox S Buyukunal C Millar AJW. Surgery for the complex Wilms tumor. Pediatr Surg Int. (2020) 36:113–27. 10.1007/s00383-019-04596-w 31701302

[68] Millar AJW Cox S Davidson A. Management of bilateral Wilms tumors. Pediatr Surg Int. (2017) 33:737–45. 10.1007/s00383-016-4047-2 28516188

[69] Grigoriev Y Lange J Peterson SM Takashima JR Ritchey ML Ko D. Treatments and outcomes for end-stage renal disease following Wilms tumor. Pediatr Nephrol. (2012) 27:1325–33. 10.1007/s00467-012-2140-x 22430485 PMC3383943

[70] Gariepy-Assal L Gilbert RD Ziaugra A Foster BJ. Management of Denys-Drash syndrome: a case series based on an international survey. Clin Nephrol Case Stud. (2018) 6:36–44. 10.5414/CNCS109515 30450273 PMC6236398

[71] Auber F Jeanpierre C Denamur E Jaubert F Schleiermacher G Patte C. Management of Wilms tumors in Drash and Frasier syndromes. Pediatr Blood Cancer. (2009) 52:55–9. 10.1002/pbc.21759 18816692

[72] Neville H Ritchey ML Shamberger RC Haase G Perlman S. Yoshioka T. The occurrence of Wilms tumor in horseshoe kidneys: a report from the national Wilms tumor study group (NWTSG). J Pediatr Surg. (2002) 37:1134–7. 10.1053/jpsu.2002.34458 12149688

[73] Sutthatarn P Gomez Quevedo O Gleason J Davidoff AM Murphy AJ. Management of intravascular thrombus in cases of bilateral Wilms tumor or horseshoe kidney. J Pediatr Surg. (2021) 5:S0022–3468. 10.1016/j.jpedsurg.2021.07.025 34452755

[74] Murphy AJ Davidoff AM. Bilateral Wilms tumor: a surgical perspective. Children. (2018) 5:134. 10.3390/children5100134 30250006 PMC6210093

[75] Wilde JC Aronson DC Sznajder B Van Tinteren H Powis M Okoye B. Nephron sparing surgery (NSS) for unilateral Wilms tumor (UWT): the SIOP 2001 experience. Pediatr Blood Cancer. (2014) 61:2175–9. 10.1002/pbc.25185 25156758

[76] Cost NG Sawicz-Birkowska K Kajbafzadeh AM Tourchi A Parigi GB Guillen G. A comparison of renal function outcomes after nephron-sparing surgery and radical nephrectomy for non-syndromic unilateral Wilms tumor. Urology. (2014) 83:1388–93. 10.1016/j.urology.2014.01.051 24768019

[77] Cozzi DA Ceccanti S Frediani S Mele E Cozzi F. Renal function adaptation up to the fifth decade after treatment of children with unilateral renal tumor: a cross-sectional and longitudinal study. Pediatr Blood Cancer. (2013) 60:1534–8. 10.1002/pbc.24545 23606234

[78] Neu MA Russo A Wingerter A Alt F Theruvath J El Malki K. Prospective analysis of long-term renal function in survivors of childhood Wilms tumor. Pediatr Nephrol. (2017) 32:1915–25. 10.1007/s00467-017-3673-9 28451896

[79] Romao RL Lorenzo AJ. Renal function in patients with Wilms tumor. Urol Oncol. (2016) 34:33–41. 10.1016/j.urolonc.2015.07.002 26278364

[80] Vanden Berg RN Bierman EN Noord MV Rice HE Routh JC. Nephron-sparing surgery for Wilms tumor: a systematic review. Urol Oncol. (2016) 34:24–32. 10.1016/j.urolonc.2015.07.003 26254695 PMC4681627

[81] Davidoff AM Giel DW Jones DP Jenkins JJ Krasin MJ Hoffer FA. The feasibility and outcome of nephron-sparing surgery for children with bilateral Wilms tumor. The St Jude children's research hospital experience: 1999–2006. Cancer. (2008) 112:2060–70. 10.1002/cncr.23406 18361398

[82] Fuchs J Schmidt A Ellerkamp V Paulsen F Melchior P Timmermann B. New aspects and innovations in the local treatment of renal and urogenital pediatric tumors. Semin Pediatr Surg. (2021) 30:151081. 10.1016/j.sempedsurg.2021.151081 34412882

[83] Aldrink JH Cost NG McLeod DJ Bates DG Stanek JR Smith EA. Technical considerations for nephron-sparing surgery in children: what is needed to preserve renal units? J Surg Res. (2018) 232:614–20. 10.1016/j.jss.2018.07.022 30463781

[84] Millar AJ Davidson A Rode H Numanoglu A Hartley PS Desai F. Nephron-sparing surgery for bilateral Wilms' tumors: a single-center experience with 23 cases. Afr J Paediatr Surg. (2011) 8:49–56. 10.4103/0189-6725.78669 21478587

[85] Duarte RJ Cristofani LM Odone Filho V Srougi M Denes FT. Videolaparoscopic radical nephrectomy after chemotherapy in the treatment of Wilms' tumor: long-term results of a pioneer group. J Pediatr Urol. (2017) 13:50.e1–50.e5. 10.1016/j.jpurol.2016.09.004 28288778

[86] Ezekian B Englum BR Gulack BC Rialon KL Kim J Talbot LJ. Comparing oncologic outcomes after minimally invasive and open surgery for pediatric neuroblastoma and Wilms tumor. Pediatr Blood Cancer. (2018) 65:e26755. 10.1002/pbc.26755 28792662

[87] Warmann SW Godzinski J van Tinteren H Heij H Powis M Sandstedt B. Minimally invasive nephrectomy for Wilms tumors in children - data from SIOP 2001. J Pediatr Surg. (2014) 49:1544–8. 10.1016/j.jpedsurg.2014.06.005 25475791

[88] Lopes RI Ming J Koyle MA Grant R Fonseca A Lorenzo AJ. Zero-Ischemia laparoscopic-assisted partial nephrectomy for the management of selected children with Wilms tumor following neoadjuvant chemotherapy. Urology. (2017) 100:103–10. 10.1016/j.urology.2016.08.051 27720972

[89] Davidoff AM Interiano RB Wynn L Delos Santos N Dome JS Green DM. Overall survival and renal function of patients with synchronous bilateral Wilms tumor undergoing surgery at a single institution. Ann Surg. (2015) 262:570–6. 10.1097/SLA.0000000000001451 26366536 PMC5187953

[90] Bouty A Blanc T Leclair MD Lavrand F Faure A Binet A. Minimally invasive surgery for unilateral Wilms tumors: multicenter retrospective analysis of 50 transperitoneal laparoscopic total nephrectomies. Pediatr Blood Cancer. (2020) 67:e28212. 10.1002/pbc.28212 32064752

[91] Blanc T Meignan P Vinit N Ballouhey Q Pio L Capito C. Robotic surgery in pediatric oncology: lessons learned from the first 100 tumors-a nationwide experience. Ann Surg Oncol. (2022) 29:1315–26. 10.1245/s10434-021-10777-6 34523002

[92] Yadav P Mahajan A Kandpal DK Chowdhary SK. Nephron-sparing surgery for syndromic Wilms' tumor: robotic approach. Urology. (2018) 116:172–5. 10.1016/j.urology.2018.03.003 29572054

[93] Meignan P Ballouhey Q Lejeune J Braik K Longis B Cook AR. Robotic-assisted laparoscopic surgery for pediatric tumors: a bicenter experience. J Robot Surg. (2018) 12:501–8. 10.1007/s11701-017-0773-2 29288372

[94] Blanc T Pio L Clermidi P Muller C Orbach D Minard-Colin V. Robotic-assisted laparoscopic management of renal tumors in children: preliminary results. Pediatr Blood Cancer. (2019) 66:3.e27867. 10.1002/pbc.27867 31136081

[95] Schmidt A Warmann SW Urla C Schaefer J Fideler F Fuchs J. Patient selection and technical aspects for laparoscopic nephrectomy in Wilms tumor. Surg Oncol. (2019) 29:14–9. 10.1016/j.suronc.2019.02.007 31196478

[96] Burnand K Roberts A Bouty A Nightingale M Campbell M Heloury Y. Laparoscopic nephrectomy for Wilms' tumor: can we expand on the current SIOP criteria? J Pediatr Urol. (2018) 14:253.e1–253.e8. 10.1016/j.jpurol.2018.01.005 29501377

[97] Hales PW Olsen OE Sebire NJ Pritchard-Jones K Clark CA. A multi-Gaussian model for apparent diffusion coefficient histogram analysis of Wilms' tumor subtype and response to chemotherapy. NMR Biomed. (2015) 28:948–57. 10.1002/nbm.3337 26058670

[98] Littooij AS Nikkels PG Hulsbergen-van de Kaa CA van de Ven CP van den Heuvel-Eibrink MM Olsen ØE. Apparent diffusion coefficient as it relates to histopathology findings in post-chemotherapy nephroblastoma: a feasibility study. Pediatr Radiol. (2017) 47:1608–14. 10.1007/s00247-017-3931-9 28669064 PMC5658478

[99] Watson T Oostveen M Rogers H Pritchard-Jones K Olsen Ø. The role of imaging in the initial investigation of pediatric renal tumors. Lancet Child Adolesc Health. (2020) 4:232–41. 10.1016/S2352-4642(19)30340-2 32007136

[100] Hötker AM Lollert A Mazaheri Y Müller S Schenk JP Mildenberger PC. Diffusion-weighted MRI in the assessment of nephroblastoma: results of a multi-center trial. Abdom Radiol. (2020) 45:3202–12. 10.1007/s00261-020-02475-w 32166338 PMC8356178

[101] Littooij AS Sebire NJ Olsen ØE. Whole-tumor apparent diffusion coefficient measurements in nephroblastoma: can it identify blastemal pre-dominance? J Magn Reson Imaging. (2017) 45:1316–24. 10.1002/jmri.25506 27726252

[102] Rogers HJ Verhagen MV Shelmerdine SC Clark CA Hales PW. An alternative approach to contrast-enhanced imaging: diffusion-weighted imaging and T_1_-weighted imaging identifies and quantifies necrosis in Wilms tumor. Eur Radiol. (2019) 29:4141–9. 10.1007/s00330-018-5907-z 30560365 PMC6610268

[103] Pachl MJ. Fluorescent guided lymph node harvest in laparoscopic Wilms nephroureterectomy. Urology. (2021) 158:189–92. 10.1016/j.urology.2021.09.015 34606881

[104] Sadeghi R Shojaeian R Hiradfar M Mohammadipour A Azadmand A Mashhadi MP. Sentinel lymph node biopsy in pediatric Wilms tumor. J Pediatr Surg. (2022) 10:S0022–3468. 10.1016/j.jpedsurg.2021.12.037 35067359

[105] Esposito C Coppola V Del Conte F Cerulo M Esposito G Farina A. Near-Infrared fluorescence imaging using indocyanine green (ICG): emerging applications in pediatric urology. J Pediatr Urol. (2020) 16:700–7. 10.1016/j.jpurol.2020.07.008 32747308

[106] Privitera L Paraboschi I Cross K Giuliani S. Above and beyond robotic surgery and 3D modeling in pediatric cancer surgery. Front Pediatr. (2021) 9:777840. 10.3389/fped.2021.777840 34988038 PMC8721224

[107] Cone EB Dalton SS Van Noord M Tracy ET Rice HE Routh JC. Biomarkers for Wilms tumor: a systematic review. J Urol. (2016) 196:1530–5. 10.1016/j.juro.2016.05.100 27259655 PMC5069120

[108] Perotti D Hohenstein P Bongarzone I Maschietto M Weeks M Radice P. Is Wilms tumor a candidate neoplasia for treatment with WNT/beta-catenin pathway modulators?-A report from the renal tumors biology-driven drug development workshop. Mol Cancer Ther. (2013) 12:2619–27. 10.1158/1535-7163.MCT-13-0335 24258344 PMC3858246

[109] Chagtai T Zill C Dainese L Wegert J Savola S Popov S. Gain of 1q as a prognostic biomarker in Wilms tumors (WTs) treated with pre-operative chemotherapy in the international society of pediatric oncology (SIOP) WT 2001 trial: a SIOP renal tumors biology consortium study. J Clin Oncol. (2016) 34:3195–203. 10.1200/JCO.2015.66.0001 27432915 PMC5505170

[110] Gratias EJ Dome JS Jennings LJ Chi YY Tian J Anderson J. Association of chromosome 1q gain with inferior survival in favorable-histology Wilms tumor: a report from the children's oncology group. J Clin Oncol. (2016) 34:3189–94. 10.1200/JCO.2015.66.1140 27400937 PMC5012705

[111] Scott RH Murray A Baskcomb L Turnbull C Loveday C Al-Saadi R. Stratification of Wilms tumor by genetic and epigenetic analysis. Oncotarget. (2012) 3:327–35. 10.18632/oncotarget.468 22470196 PMC3359888

[112] Ruteshouser EC Robinson SM Huff V. Wilms tumor genetics: mutations in WT1, WTX, and CTNNB1 account for only about one-third of tumors. Genes Chromosomes Cancer. (2008) 47:461–70. 10.1002/gcc.20553 18311776 PMC4332772

[113] Breslow NE Norris R Norkool PA Kang T Beckwith JB Perlman EJ. Characteristics and outcomes of children with the Wilms tumor-Aniridia syndrome: a report from the national Wilms tumor study group. J Clin Oncol. (2003) 21:4579–85. 10.1200/JCO.2003.06.096 14673045

[114] Pelletier J Bruening W Kashtan CE Mauer SM Manivel JC Striegel JE. Germline mutations in the Wilms' tumor suppressor gene are associated with abnormal urogenital development in Denys-Drash syndrome. Cell. (1991) 67:437–47. 10.1016/0092-8674(91)90194-4 1655284

[115] Brok J Treger TD Gooskens SL van den Heuvel-Eibrink MM Pritchard-Jones K. Biology and treatment of renal tumors in childhood. Eur J Cancer. (2016) 68:179–95. 10.1016/j.ejca.2016.09.005 27969569

[116] Gadd S Huff V Walz AL Ooms A Armstrong AE Gerhard DS. A children's oncology group and TARGET initiative exploring the genetic landscape of Wilms tumor. Nat Genet. (2017) 49:1487–94. 10.1038/ng.3940 28825729 PMC5712232

[117] Wegert J Ishaque N Vardapour R Georg C Gu Z Bieg M. Mutations in the SIX1/2 pathway and the DROSHA/DGCR8 miRNA microprocessor complex underlie high-risk blastemal type Wilms tumors. Cancer Cell. (2015) 27:298–311. 10.1016/j.ccell.2015.01.002 25670083

[118] Walz AL Ooms A Gadd S Gerhard DS Smith MA Guidry Auvil JM. Recurrent DGCR8, DROSHA, and SIX homeodomain mutations in favorable histology Wilms tumors. Cancer Cell. (2015) 27:286–97. 10.1016/j.ccell.2015.01.003 25670082 PMC4800737

[119] Cresswell GD Apps JR Chagtai T Mifsud B Bentley CC Maschietto M. Intra-tumor genetic heterogeneity in Wilms tumor: clonal evolution and clinical implications. EBioMedicine. (2016) 9:120–9. 10.1016/j.ebiom.2016.05.029 27333041 PMC4972528

[120] Perlman EJ Grundy PE Anderson JR Jennings LJ Green DM Dome JS. WT1 mutation and 11P15 loss of heterozygosity predict relapse in very low-risk Wilms tumors treated with surgery alone: a children's oncology group study. J Clin Oncol. (2011) 29:698–703. 10.1200/JCO.2010.31.5192 21189373 PMC3056654

[121] Bjornsson HT Brown LJ Fallin MD Rongione MA Bibikova M Wickham E. Epigenetic specificity of loss of imprinting of the IGF2 gene in Wilms tumors. J Natl Cancer Inst. (2007) 99:1270–3. 10.1093/jnci/djm069 17686827 PMC5533193

[122] Wegert J Wittmann S Leuschner I Geissinger E Graf N Gessler M. inactivation is a frequent, but late event in Wilms tumors without apparent clinical impact. Genes Chromosomes Cancer. (2009) 48:1102–11. 10.1002/gcc.20712 19760609

[123] Perlman EJ Gadd S Arold ST Radhakrishnan A Gerhard DS Jennings L. MLLT1 YEATS domain mutations in clinically distinctive favorable histology Wilms tumors. Nat Commun. (2015) 6:10013. 10.1038/ncomms10013 26635203 PMC4686660

[124] Williams RD Chagtai T Alcaide-German M Apps J Wegert J Popov S. Multiple mechanisms of MYCN dysregulation in Wilms tumor. Oncotarget. (2015) 6:7232–43. 10.18632/oncotarget.3377 25749049 PMC4466681

[125] Armstrong AE Gadd S Huff V Gerhard DS Dome JS Perlman EJ. unique subset of low-risk Wilms tumors is characterized by loss of function of TRIM28 (KAP1), a gene critical in early renal development: a children's oncology group study. PLoS ONE. (2018) 13:e0208936. 10.1371/journal.pone.0208936 30543698 PMC6292605

[126] Ooms AH Gadd S Gerhard DS Smith MA Guidry Auvil JM Meerzaman D. Significance of TP53 mutation in Wilms tumors with diffuse anaplasia: a report from the children's oncology group. Clin Cancer Res. (2016) 22:5582–91. 10.1158/1078-0432.CCR-16-0985 27702824 PMC5290091

[127] Hanks S Perdeaux ER Seal S Ruark E Mahamdallie SS Murray A. Germline mutations in the PAF1 complex gene CTR9 pre-dispose to Wilms tumor. Nat Commun. (2014) 5:4398. 10.1038/ncomms5398 25099282 PMC4143912

[128] Capasso M Montella A Tirelli M Maiorino T Cantalupo S Iolascon A. Genetic pre-disposition to solid pediatric cancers. Front Oncol. (2020) 10:590033. 10.3389/fonc.2020.590033 33194750 PMC7656777

[129] Kratz CP Jongmans MC Cave H Wimmer K Behjati S Guerrini-Rousseau L. Pre-disposition to cancer in children and adolescents. Lancet Child Adolesc Health. (2021) 5:142–54. 10.1016/S2352-4642(20)30275-3 33484663

[130] Mahamdallie S Yost S Poyastro-Pearson E Holt E Zachariou A Seal S. Identification of new Wilms tumor pre-disposition genes: an exome sequencing study. Lancet Child Adolesc Health. (2019) 3:322–31. 10.1016/S2352-4642(19)30018-5 30885698 PMC6472290

[131] Vujanic GM Kelsey A Mitchell C Shannon RS Gornall P. The role of biopsy in the diagnosis of renal tumors of childhood: results of the UKCCSG Wilms tumor study 3. Med Pediatr Oncol. (2003) 40:18–22. 10.1002/mpo.10216 12426681

[132] Treger TD Chagtai T Butcher R Cresswell GD Al-Saadi R Brok J. Somatic TP53 mutations are detectable in circulating tumor DNA from children with anaplastic Wilms tumors. Transl Oncol. (2018) 11:1301–6. 10.1016/j.tranon.2018.08.006 30172241 PMC6121832

[133] Weiser DA West-Szymanski DC Fraint E Weiner S Rivas MA Zhao CWT. Progress toward liquid biopsies in pediatric solid tumors. Cancer Metastasis Rev. (2019) 38:553–71. 10.1007/s10555-019-09825-1 31836951 PMC6995761

[134] Apple A Lovvorn HN 3rd. Wilms tumor in Sub-Saharan Africa: molecular and social determinants of a global pediatric health disparity. Front Oncol. (2020) 10:606380. 10.3389/fonc.2020.606380 33344257 PMC7746839

[135] Fukuzawa R Breslow NE Morison IM Dwyer P Kusafuka T Kobayashi Y. Epigenetic differences between Wilms' tumors in white and east-Asian children. Lancet. (2004) 363:446–51. 10.1016/S0140-6736(04)15491-3 14962525

[136] Haruta M Arai Y Watanabe N Fujiwara Y Honda S Ohshima J. Different incidences of epigenetic but not genetic abnormalities between Wilms tumors in Japanese and Caucasian children. Cancer Sci. (2012) 103:1129–35. 10.1111/j.1349-7006.2012.02269.x 22409817 PMC7685077

[137] Girón-Vallejo Ó García-Calderón D Ruiz-Pruneda R Cabello-Laureano R Doménech-Abellán E Fuster-Soler JL. Three-dimensional printed model of bilateral Wilms tumor: a useful tool for planning nephron sparing surgery. Pediatr Blood Cancer. (2018) 65:e26894. 10.1002/pbc.26894 29230948

[138] Kalapurakal JA Lee B Bautista J Rigsby C Helenowski I. Gopalakrishnan M. Cardiac-sparing whole lung intensity modulated radiation therapy in children with Wilms tumor: final report on technique and abdominal field matching to maximize normal tissue protection. Pract Radiat Oncol. (2019) 9:e62–73. 10.1016/j.prro.2018.07.005 30096378

[139] Vogel J Lin H Both S Tochner Z Balis F Hill-Kayser C. Pencil beam scanning proton therapy for treatment of the retroperitoneum after nephrectomy for Wilms tumor: a dosimetric comparison study. Pediatr Blood Cancer. (2017) 64:39–45. 10.1002/pbc.26176 27565764

